# Colon Cancer-Derived Exosomal LncRNA-XIST Promotes M2-like Macrophage Polarization by Regulating PDGFRA

**DOI:** 10.3390/ijms252111433

**Published:** 2024-10-24

**Authors:** Beibei Gao, Li Wang, Ting Wen, Xiaoge Xie, Xiaoyi Rui, Qiaoyi Chen

**Affiliations:** Department of Cell Biology and Genetics, School of Basic Medical Sciences, Xi’an Jiaotong University, Xi’an 710049, China; gaobeibei@stu.xjtu.edu.cn (B.G.); wangli@stu.xjtu.edu.cn (L.W.); 3120115010@stu.xjtu.edu.cn (T.W.); xiexiaoge@stu.xjtu.edu.cn (X.X.); ruixiaoyi@stu.xjtu.edu.cn (X.R.)

**Keywords:** macrophage, polarization, exosomes, LncXIST, PDGFRA, miR-17-5p

## Abstract

Colon cancer ranks second in overall cancer-related deaths and poses a serious risk to human life and health. In recent years, exosomes are believed to play an important and significant role in cancer, especially tumor-derived exosomes (TDEs). Previous studies have highlighted the pivotal role of exosomes in tumor development, owing to their ability to mediate communication between tumor cells and macrophages, induce macrophage M2 polarization, and facilitate the progression of tumorigenesis. In this study, we revealed that colon cancer-derived exosomes promoted M2-like macrophage polarization. Moreover, exosome-induced M2-like macrophages, in turn, promoted the proliferation, migration, and invasion abilities of colon cancer cells. Specifically, CT26- and HCT116-derived exosomes led to the activation of AKT, ERK, and STAT3/6 signaling pathways in THP-1(Mφ) cells. Furthermore, our findings showed that colon cancer-derived exosomes secreted lncXIST to sponge miR-17-5p, which, in turn, promoted the expression of PDGFRA, a common gene found in all three signaling pathways, to facilitate M2-like macrophage polarization. Dual-luciferase reporter assays confirmed the binding relationship between lncXIST and miR-17-5p, as well as miR-17-5p and PDGFRA. Collectively, our results highlight the novel role of lncXIST in facilitating macrophage polarization by sponging miR-17-5p and regulating PDGFRA expression.

## 1. Introduction

Colon cancer is the third most common malignant tumor worldwide. The global disease burden is projected to increase to 3.2 million new cases and 1.6 million deaths by 2040. The shift toward earlier age of onset and increased probability of being diagnosed in people under the age of 50 is contributed by a mixture of genetic, lifestyle, and environmental risk factors. Endoscopic therapy, surgical therapy, adjuvant chemotherapy, and targeted therapy are common treatments for colon cancer. Immunotherapy can be used for early-stage colon cancer, and both overall endoscopic mucosal resection and endoscopic submucosal resection can be performed via endoscopy to remove large and complex lesions. Despite available treatment methods, the five-year survival rate and prognosis of colon cancer remain suboptimal, thus highlighting the critical need for understanding the underlying molecular mechanisms for advancing early diagnosis and treatment of colon cancer.

Recently, an increasing number of reports have shown that the tumor microenvironment (TME) is closely associated with tumor development and treatment outcomes. The TME is composed of a complex junction of diverse cell types including tumor cells, immune cells, endothelial cells, peripheral blood vessels, extracellular matrix, and cancer-associated fibroblasts [1,2]. These cells constantly interact and modify the environment in which the tumor survives. Among the varied cell types, macrophages hold an irreplaceable role in the TME. On the one hand, macrophages are the most abundant stromal cells and the most critical immune cells in the TME to combat tumor growth. On the other hand, the polarization of macrophages can lead them to play entirely opposite roles in tumor. The plasticity and heterogeneity of macrophages is reflected by the fact that the macrophage phenotype and function are regulated by the surrounding environment, enabling them to switch from one phenotype to another. Notably, macrophages found in the TME are known as tumor-associated macrophages (TAMs). The phenotype of TAMs changes under the influence of the microenvironment. Specifically, macrophages are classified into two primary phenotypes: pro-inflammatory/anti-tumor type M1 and anti-inflammatory/pro-tumor type M2. While TAMs generally appear as M1-type in the early stage, they can transform into M2-type under the influence of the TME to facilitate tumorigenesis [3,4,5,6]. M2 macrophages are known to promote an immune-suppressive TME and enhance tumor-cell survival and metastasis. Thus, investigating the molecular switch behind the temporal differentiation of TAMs is imperative for developing effective therapeutic strategies for inhibiting tumor growth.

In this study, we focused on the crosstalk between colon cancer-derived exosomes and TAMs. Extracellular vesicles (EVs) are microscopic vesicles secreted by cells, previously thought to merely function as cargos for excreting waste products. Advanced understanding has revealed that EVs are important cell–cell communication vectors, and carry numerous cellular components including proteins, lipids, polysaccharides, metabolites, DNA, and RNA [7,8]. EVs include microvesicles, exosomes, ectosomes, oncosomes, and cytoplasts [9]. Exosomes are the smallest class of EVs, and are about 40~180 nm [10]. In recent years, tumor-derived exosomes (TDEs) are thought to play an important role in cancer. Specifically, TDEs have been demonstrated to contribute to macrophage heterogeneity by carrying and delivering a variety of RNAs, including microRNA (miRNA), long non-coding RNA (lncRNA), and circular RNA (circRNA) [11,12,13]. For instance, Chen et al. found that lung adenocarcinoma cells transferred miR-19b-3p to macrophages via exosomes, which downregulated PTPRD and activated STAT3, resulting in the M2 polarization of macrophages [14]. Moreover, Xiang et al. found that ovarian cancer-derived exosomes enriched with miR-222-3p downregulated SOCS3 and induced STAT3 activation, which resulted in the acquisition of M2 phenotype in macrophages [15]. In contrast to direct miRNA-induced mRNA degradation, lncRNAs and cirRNAs are known to indirectly influence gene expression by sponging target miRNAs. Unlike miRNAs with their high expression levels and short sequences, lncRNAs are highly heterogeneous primary sequences of more than 200 nucleic acids in length, once considered transcriptional noise due to their lack of protein-coding ability [16]. LncRNAs have enhancer-like properties and can interact with miRNAs, mRNAs, RNA-binding proteins, and transcription factors [17,18]. LncRNAs can also stabilize mRNA translation and act as precursors to miRNAs [18]. More important, lncRNAs can act as competing endogenous RNAs (ceRNAs) to target and absorb miRNAs like sponges. The role of lncRNAs in tumorigenesis and macrophage polarization has also been reported. A study led by Zhang et al. showed that renal cell carcinoma-derived exosomes can transfer lncARSR to sponge miR-34/miR-449 and increase STAT3 expression in macrophages, thereby promoting M2-like macrophage polarization [19]. Moreover, Huang et al. found that exosomal circSAFB2 is able to sponge miR-620 and activate the JAK1/STAT3 pathway as means of mediating M2 macrophage polarization [20]. Notably, among the various signaling pathways reportedly involved in macrophage polarization, those involving PI3K/AKT, JAK/STAT, and MAPK/ERK are especially prominent [12,21,22]. In colon cancer, the upregulation of lncXIST has been demonstrated to sponge miR-34a and activate the Wnt/β-catenin pathway to promote colon cancer development [23]. However, it is unclear whether lncXIST plays a role in macrophage polarization. In this study, we investigated the molecular mechanism of colon cancer-derived exosomes and showed that lncXIST is able to mediate M2 macrophage polarization by regulating miR-17-5p and PDGFRA.

## 2. Results

### 2.1. Colon Cancer Cell-Derived Exosomes Promote M2-like Macrophage Polarization

**Figure 1:**
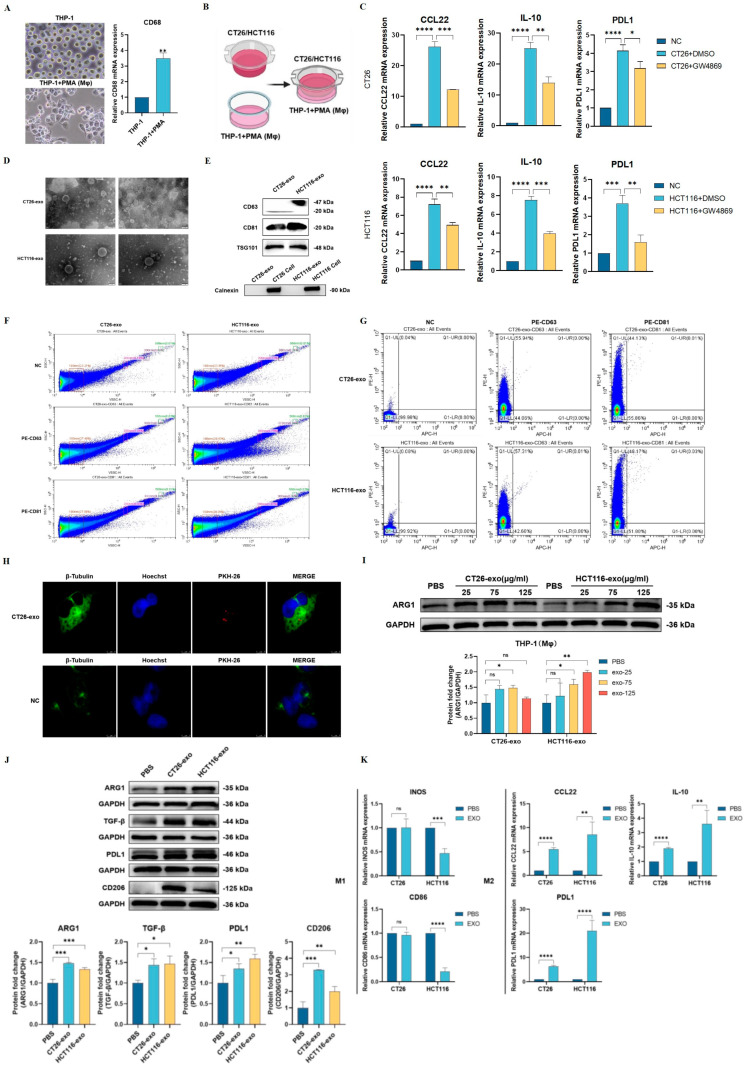
Colon cancer cell- derived exosomes promote M2-like macrophage polarization. (**A**) THP-1 cells were induced into macrophages (Mφ) via PMA treatment, and the expression level of CD68 was detected by qRT-PCR (*n* = 3). (**B**) Schematic diagram illustrating the use of Transwell system to co-culture CT26/HCT116 and THP- 1 (Mφ) cells. (**C**) qRT-PCR was performed to detect the mRNA levels of M2 markers (CCL22, IL10, and PDL1) in THP-1 (Mφ) cells. The negative control (NC) group represents THP-1 cells induced with PMA alone, and the experimental groups were DMSO-treated CT26 or HCT116 cells (CT26/HCT116 + DMSO) and GW4869-treated CT26 or HCT116 cells (CT26/HCT116 + GW4869) (*n* = 3). (**D**) Transmission electron microscopy revealed that the extracted particles were around 100 nm with round morphology and intact double-layer membrane structure. (**E**) Western blotting results showed that the extracted exosomes from CT26 and HCT116 cells were highly expressed in CD63, CD81, and TSG101, but not the endoplasmic reticulum protein Calnexin. (**F**,**G**) CytoFLEX flow cytometer was used to detect vesicles of 100 nm in size. (**F**) Exosomes were labeled either NC, PE-CD63, or PE-CD81. A total of 23.21% (CT26) and 23.85% (HCT116) of extracted exosomes were 100 nm in size. (**G**) The expression of CD63 and CD81 was detected in both CT26- and HCT116-derived exosomes. A total of 55.94% of PE-CD63 and 44.13% of PE-CD81 was characterized in CT26-derived exosomes. A total of 57.31% of PE-CD63 and 48.17% of PE-CD81 was characterized in HCT116-derived exosomes. (**H**) CT26-derived exosomes were labeled using PKH-26 (red) and co-incubated with THP-1 (Mφ) cells. The nuclei and cytoskeleton of macrophages were sequentially labeled with Hoechst (blue) and β-Tubulin (green), respectively. After 24 h, red fluorescent dots were subsequently observed within the cytoskeleton by laser confocal microscopy, suggesting that PKH-26-labeled colon cancer cell-derived exosomes could be taken up by macrophages. (**I**) To determine the optimal concentration of CT26-exo and HCT116-exo necessary for macrophage polarization, various concentrations (25 μg/mL, 75 μg/mL, and 125 μg/mL) ofCT26-exo and HCT116-exo were added to THP-1 (Mφ). Western blotting results showed that the optimal concentration of CT26-exo for promoting ARG1 was 75 μg/mL, while the optimal concentration of HCT116-exo was 125 μg/mL. (**J**) Western blotting results indicated that THP-1 (Mφ) cells treated with CT26-exo or HCT116-exo showed markedly increased protein levels of ARG1, TGF-β, PDL1, and CD206 compared to the PBS-treated group. Quantification analysis is shown below. (**K**) qRT-PCR results showed that THP-1 (Mφ) cells treated with CT26-exo demonstrated increased M2 markers CCL22, IL-10, and PDL1. M1 markers INOS and CD86 were reduced while M2 markers CCL22, IL-10, and PDL-1 were increased in HCT116-exo-treated THP-1 (Mφ) cells. All experiments were performed in triplicates, * denotes *p* < 0.05, ** denotes *p* < 0.01, *** denotes *p* < 0.001, and **** indicates *p* < 0.0001.

Our study began by investigating the effect of colon cancer cells on the direction of macrophage polarization and whether exosomes played a role in the process. First, human monocytic-leukemia (THP-1) cells were induced into macrophages (Mφ) via PMA treatment (Figure 1A). Under the stimulatory effect of PMA, the morphology of THP-1 cells conformed to the characteristics of macrophages (Mφ). At the mRNA level, PMA stimulation significantly elevated the expression of CD68, a marker for macrophages (Mφ) (Figure 1A). Subsequently, CT26 or HCT116 cells were treated with either DMSO or GW4869 (exosome inhibitor), and co-cultured with THP-1 (Mφ) cells through an in vitro transwell system for 24 h (Figure 1B). THP-1cells treated with PMA alone served as the control group. M2 macrophage markers including CCL22, IL10, and PDL1 were then determined for each group using qRT-PCR. As shown in Figure 1C, THP-1 (Mφ) cells co-cultured with CT26 + DMSO or HCT116 + DMSO showed significantly upregulated M2 polarization makers compared to the control group, indicating that both CT26 and HCT116 cells can promote M2 polarization. On the other hand, THP-1 (Mφ) cells co-cultured with CT26 + GW4869 or HCT116 + GW4869 led to a reduction in M2 markers (Figure 1C), suggesting that the effect of CT26 and HCT116 cells on macrophage M2 polarization is dependent on exosome mediation.

To examine the function of cancer cell-derived exosomes on macrophage polarization, we first extracted exosomes from CT26 and HCT116 cells through ultracentrifugation. The extracted exosomes were then confirmed and characterized via transmission electron microscopy (Figure 1D) and Western blotting analyses (Figure 1E). The particle size was determined using CytoFLEX flow cytometer. As shown in Figure 1E, the extracted exosomes showed high protein levels of CD81 and TSG101, but not Calnexin. Flow cytometry assay revealed that the extracted exosomes were concentrated at 100 nm (Figure 1F) and that both CD63 and CD81 were detected in CT26- and HCT116-derived exosomes (Figure 1G). Together, these results indicate that the extracted exosomes are in line with expected extraction criteria [24]. In an effort to confirm that colon cancer-derived exosomes are taken up by macrophages, PKH-26 (red) was used to label the exosomes, which were then co-incubated with THP-1 (Mφ) macrophages. After 24 h, the nuclei of macrophages were stained with Hoechst (blue), and the cytoskeleton of macrophages was stained with β-Tubulin (green), and, subsequently, visualized under a laser confocal microscope. As shown in Figure 1H, red fluorescent dots were observed around the nucleus and within the cytoskeleton of THP-1 (Mφ) macrophages, indicating that PKH-26-labeled exosomes of colon cancer cell origin can be taken up by macrophages.

To verify that colon cancer cell-derived exosomes are responsible for M2 polarization, we subjected THP-1 (Mφ) cells under the treatment of either PBS, CT26-, or HCT116-derived exosomes. The optimal concentration of exosomes necessary for inducing M2 polarization for CT26-exosomes was 75 µg/mL, and 125 µg/mL for HCT116-exosomes (Figure 1I). Western blotting analyses of M2 phenotypic markers (ARG1, TGF-β, PDL1, and CD206), as well as M1 phenotypic makers (INOS and CD86) were then determined. As indicated in Figure 1J, treatment with CT26-exo and HCT116-exo significantly upregulated the expression of ARG1, TGF-β, PDL1, and CD206 in macrophages compared with the control group. In addition, qRT-PCR results indicated that the HCT116-exo group demonstrated increased mRNA expressions of CCL22, IL10, and PDL1(M2 markers), and reduced expression of NOS and CD86 (M1 markers) (Figure 1K). While the CT26-exo group also showed increased M2 markers, the changes in INOS and CD86 mRNA levels were not significant. This suggests that CT26-exo may have a more significant effect on markers promoting M2-like macrophages rather than those promoting M1 macrophages. Overall, the results from this section indicate that CT26 and HCT116 cancer cells promote macrophage M2 polarization through secreted exosomes.

### 2.2. Polarized M2 Macrophages Promote Colon Cancer Cell Proliferation, Migration, and Invasion

**Figure 2:**
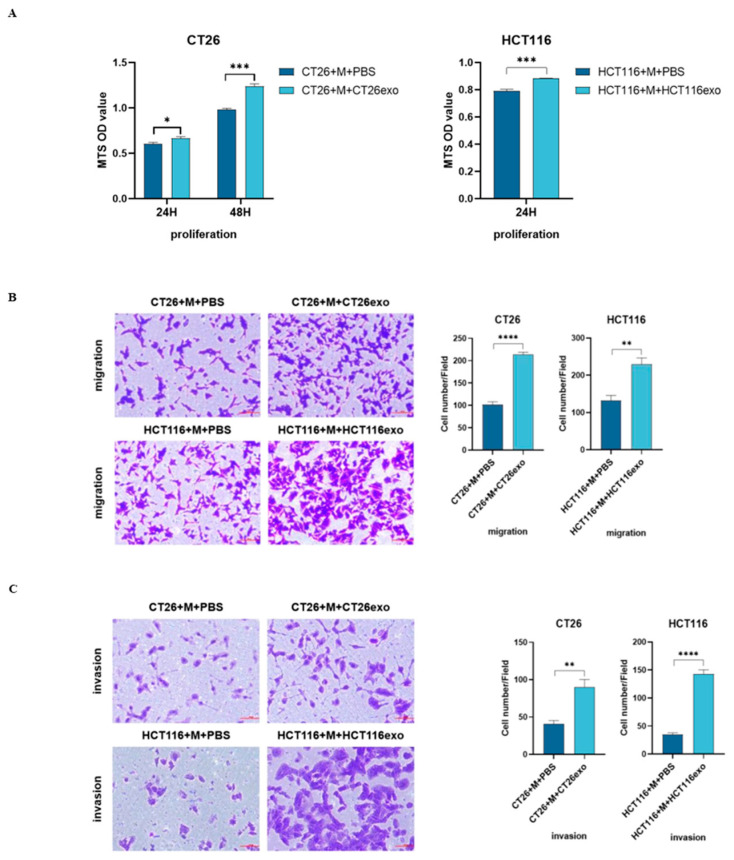
Polarized M2 macrophages promote colon cancer cell proliferation, migration, and invasion. (**A**) For the experimental groups, CT26-exo and HCT116-exo-treated THP-1 (Mφ) cell medium was collected and cultured with CT26 and HCT116 cells, respectively. The control group was cultured with PBS-treated THP-1 (Mφ) cell medium. MTS results indicated that the experimental groups showed significantly increased cell viability compared to the control group. (**B**,**C**) Transwell migration assay showed that both CT26 and HCT116 cells maintained in CT26-exo or HCT116-exo-treated THP-1 (Mφ) cell medium showed increased number of (**B**) migrating and (**C**) invading cells. All experiments were performed in triplicates, * denotes *p* < 0.05, ** denotes *p* < 0.01, *** denotes *p* < 0.001, and **** indicates *p* < 0.0001.

M2-type macrophages are known to facilitate and promote cancer cell growth. To examine the function of CT26-exo and HCT116-exo-induced M2 macrophages, we treated CT26 and HCT116 cells with M2 macrophage medium for 24 h. MTS assay results showed that M2 macrophage medium-treated CT26 and HCT116 cells demonstrated significantly increased viability compared to the control group (Figure 2A). However, although this change is significant, the effect is minor. Moreover, Transwell assay results showed that CT26 and HCT116 cells treated with M2 macrophage medium both demonstrated an increased number of migrating cells compared to the control group (Figure 2B). Moreover, invasion assay showed that CT26 and HCT116 cells treated with M2 macrophage medium resulted in an increased number of invasive cells compared to the control group (Figure 2C). Together, these results suggest that M2 polarized macrophages induced by CT26 and HCT116 cells can, in turn, facilitate the abilities of tumor cells to proliferate, migrate, and invade.

### 2.3. Colon Cancer Cell-Derived Exosomes Promote AKT-, ERK-, and STAT3/6-Related Signaling Pathways

**Figure 3:**
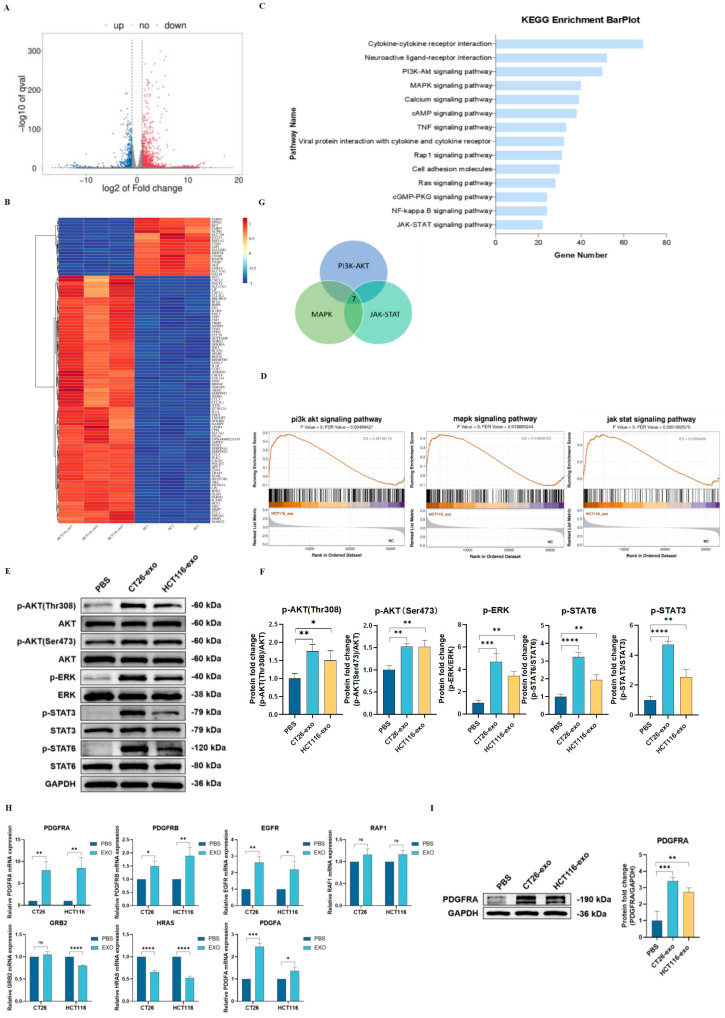
Colon cancer cell-derived exosomes promote AKT-, ERK-, and STAT3/6-related signaling pathways. (**A**) RNA-seq analysis was performed using HCT116-exo-treated THP-1(Mφ) cells and PBS-treated THP-1(Mφ) cells as the control group. Volcano plot showed a total of 1944 significantly differentially expressed genes were found between the two groups, including 1350 upregulated genes and 594 downregulated genes. (**B**) Heatmap of top 100 differentially expressed genes. (**C**) KEGG analysis showed enrichment in multiple signaling pathways, including PI3K-AKT, MAPK, and JAK-STAT pathways, which are closely associated with M2-like macrophage polarization. (**D**) GSEA enrichment analysis showed enrichment in PI3K/AKT, MAPK, and JAK/STAT signaling pathways. (**E**) Western blotting analysis showed that THP-1 (Mφ) cells treated with CT26-exo and HCT116-exo showed significantly higher protein levels of p-AKT (Thr308), p-AKT (Ser473), p-ERK, p-STAT3, and p-STAT6 compared with PBS-treated control group. (**F**) Quantification of Western blotting analysis showed increased p-AKT (Thr308)/AKT, p-AKT (Ser473)/AKT, p-ERK/ERK, p-STAT3/STAT3, and pSTAT6/STAT6 ratios in THP-1 (Mφ) cells treated with CT26-exo and HCT116-exo compared to the control group. (**G**) Venn diagram showed that seven genes (PDGFRA, PDGFRB, EGFR, RAF1, HRAS, GRB2, and PDGFA) were found in common between PI3K/AKT, MAPK, and JAK/STAT signaling pathways based on the sequencing results. (**H**) qRT-PCR was performed to validate the mRNA expression level of the selected genes. PDGFRA expression was most significantly upregulated in CT26-exo and HCT116-exo-treated THP-1 (Mφ) cells compared to the control group. (**I**) Western blotting analysis showed that THP-1 (Mφ) cells treated with CT26-exo and HCT116-exo demonstrated increased protein levels of PDGFRA compared to the control group. Quantification of Western blotting results shown right. All experiments were performed in triplicates, * denotes *p* < 0.05, ** denotes *p* < 0.01, *** denotes *p* < 0.001, and **** indicates *p* < 0.0001.

In effort to elucidate the mechanism underlying colon cancer exosome-induced M2 polarization, RNA-seq was performed using PBS-treated (control) and HCT116 exosome-treated THP-1 (Mφ) cells. A total of 1944 differentially expressed genes (DEGs) were found between the two groups, including 1350 upregulated and 594 downregulated genes (Figure 3A,B). In addition, both KEGG and GSEA pathway enrichment analyses showed an enrichment in PI3K-AKT, MAPK/ERK, and JAK-STAT signaling pathways (Figure 3C,D). Noted, these three pathways have all been previously shown to be implicated in macrophage M2 polarization [14,22,25,26]. Interestingly, the Western blotting results showed that THP-1(Mφ) cells treated with either CT26-exo or HCT116-exo demonstrated significantly higher levels of p-AKT(Thr308)/AKT, p-AKT(Ser473)/AKT, p-ERK/ERK, p-STAT3/STAT3, and p-STAT6/STAT6 ratios (Figure 3E,F). These results suggest that CT26- and HCT116-derived exosomes may promote M2 macrophage polarization through the activation of all three signaling pathways rather than relying on a singular pathway. Next, from the sequencing results, we screened and selected seven genes (PDGFRA, PDGFRB, EGFR, RAF1, HRAS, GRB2, and PDGFA) that are collectively involved in all three signaling pathways (Figure 3G). As shown through qRT-PCR analyses, of these seven genes, only PDGFRA, PDGFRB, EGFR, and HRAS showed significant changes in the same direction as the RNA-Seq results. Notably, PDGFRA showed the highest fold change compared to the other three genes. Moreover, through Western blotting analyses, we showed that THP-1(Mφ) cells treated with CT26- and HCT116-derived exosomes also showed significantly higher PDGFRA protein levels (Figure 3I). Collectively, our data suggest that PDGFRA may be an important actor in promoting CT26- and HCT116-induced M2 macrophage polarization, specifically through the activation of AKT, ERK, and STAT3/6 signaling pathways.

### 2.4. LncXIST Is Highly Expressed in Colon Cancer Cells and Cancer Exosome-Treated THP-1(Mφ) Cells

**Figure 4:**
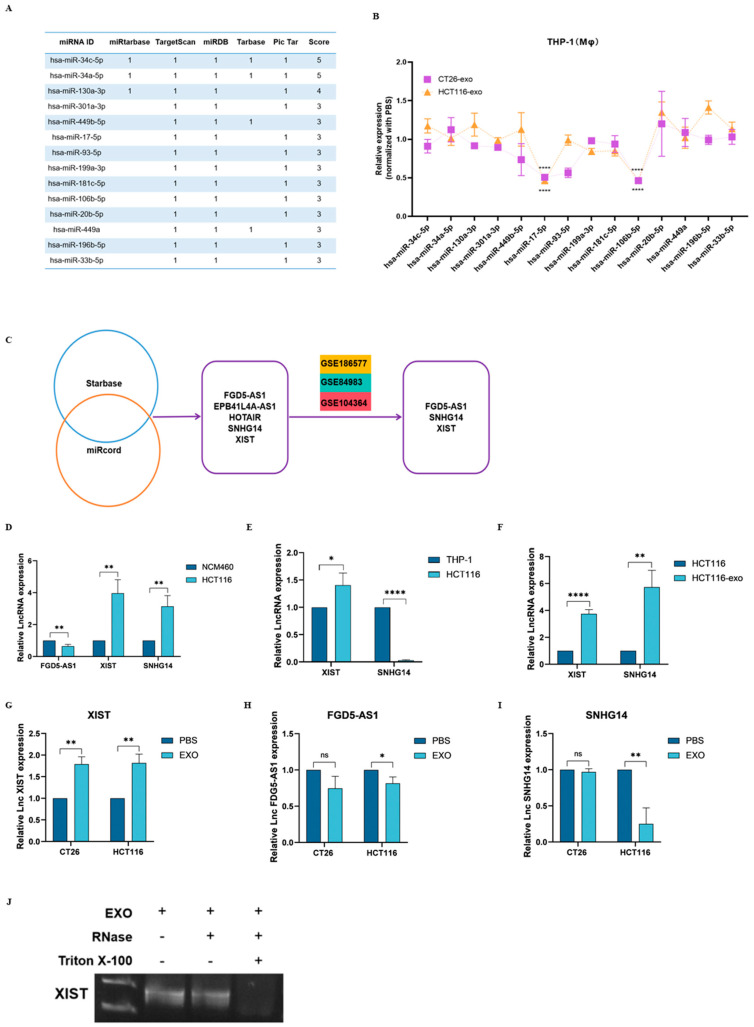
LncXIST is highly expressed in colon cancer cells and cancer exosome-treated THP-1(Mφ) cells. (**A**) PDGFRA-binding miRNAs were predicted using miRtarbase, TargetScan, miRDB, Tarbase, and Pic Tar databases. (**B**) Fourteen candidate miRNAs with high scores were selected for subsequent qRT-PCR validation. The results, normalized to the PBS-treated group, indicated that THP-1 (Mφ) cells treated with CT26-exo and HCT116-exo showed significantly reduced miR-17-5p and miR-106b-5p expression levels. (**C**) Five candidate miR-17-5p-targeting lncRNAs were predicted using the Starbase and miRcord databases. Three of the five candidate lncRNAs were also found highly expressed in colon cancer-related GEO databases (GSE186577, GSE84983 and GSE104364). (**D**) qRT-PCR analysis showed that compared with normal colon epithelial NCM460 cells, HCT116 cells showed significantly higher expression of lncXIST and lncSNHG14, and lower expression of lncFGD5-AS1. (**E**) qRT-PCR analysis showed that compared with THP-1 cells, HCT116 cells showed significantly higher expression of lncXIST but lower expression of lncSNHG14. (**F**) qRT-PCR analysis showed that compared with HCT116 cells, HCT116-derived exosomes showed significantly higher expression of both lncXIST and lncSNHG. (**G**–**I**) qRT-PCR analysis showed that only lncXIST was highly expressed in THP-1 (Mφ) cells treated with both CT26-exo and HCT116-exo compared to the PBS control group, but not FGD5-AS1 (**H**) nor SHNG14 (**I**). (**J**) Agarose gel electrophoresis analysis showed the degradation of lncXIST when treated with both RNase and Triton X-100. All experiments were performed in triplicates, ns denotes no-significance, * denotes *p* < 0.05, ** denotes *p* < 0.01, and **** indicates *p* < 0.0001.

Having confirmed that THP-1(Mφ) cells treated with CT26- and HCT116-derived exosomes show elevated PDGFRA mRNA and protein levels, we next searched for potential upstream regulators. First, through miRtarbase, TargetScan, miRDB, Tarbase, and Pic Tar database screening and overlapping analyses, 14 candidate miRNAs were chosen for further verification by qRT-PCR (Figure 4A). As our results indicated, THP-1 (Mφ) cells treated with CT26-exo and HCT116-exo show significantly reduced miR-17-5p and miR-106b-5p levels (Figure 4B). Interestingly, miR-17-5p and miR-106b-5p belong to the same family; thus, only miR-17-5p was selected for subsequent verifications. As previously mentioned, lncRNAs can bind and absorb target miRNAs, thereby indirectly regulating downstream gene expression. To identify potential upstream lncRNAs that specifically target miR-17-5p, we first screened for candidate lncRNAs using Starbase and miRcord databases, which included lncFGD5-AS1, lncEPB41L4A-AS1, lncHOTAIR, lncSNHG14, and lncXIST. Next, through colon cancer GEO database screening (GSE186577, GSE84983, and GSE104364), we found that, of the five candidate lncRNAs, lncFGD5-AS1, lncSNHG14, and lncXIST were found to be highly expressed in colon cancer patients (Figure 4C). Subsequently, we examined the expression of the three candidate lncRNAs through qRT-PCR. As shown in Figure 4D, compared to normal colon epithelial NCM460 cells, HCT116 cells showed significantly upregulated lncXIST and lncSNHG14 mRNA levels. When compared with THP-1(Mφ) cells, only lncXIST was found to be significantly upregulated in HCT116 cells (Figure 4E). Interestingly, both lncXIST and lncSNHG14 were found to be higher in HCT116-exosomes compared to HCT116 cells (Figure 4F). Next, we examined whether treatment with colon cancer cell-derived exosomes can promote candidate lncRNA upregulation in THP-1(Mφ) cells. As indicated in Figure 4G–I, CT26-exo and HCT116-exo-treated THP-1(Mφ) cells showed significantly upregulated lncXIST, but not lncFGD5-AS1 nor lncSNHG14 mRNA levels. Collectively, these results indicate that, as a potential upstream regulator of miR-17-5p, lncXIST is found to be highly expressed in colon cancer cells and colon cancer cell-derived exosomes. Moreover, treatment with CT26-exo and HCT116-exo led to significantly elevated lncXIST levels in THP-1(Mφ) cells. To further determine whether lncXIST is encapsulated in exosomes, agarose gel electrophoresis was performed. As shown in Figure 4J, lncXIST was detected in both HCT116-derived exosomes as well as RNase-treated HCT116exosomes. However, degradation of lncXIST was evident when treated with both RNase and Triton X-100, suggesting that lncXIST is stabilized under exosome encapsulation, and was degraded only after disruption of the exosome membrane in the presence of Triton X-100.

### 2.5. miR-17-5p Overexpression Inhibits lncXIST-Induced PDGRA Expression and M2 Macrophage Polarization

**Figure 5:**
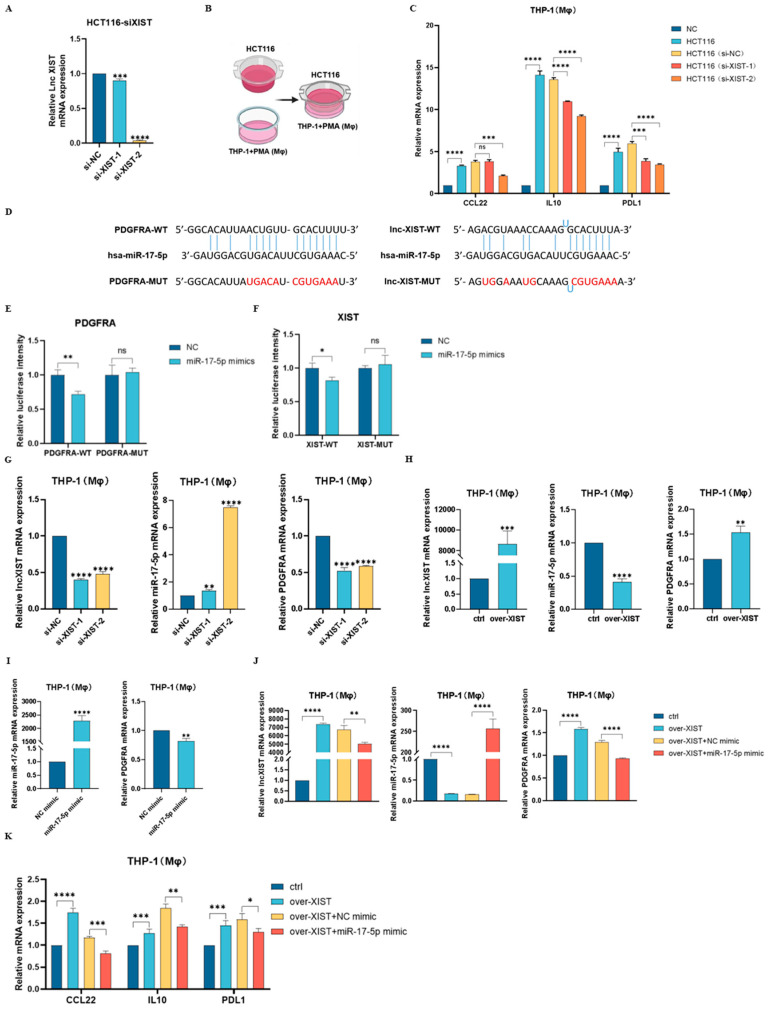
miR-17-5p overexpression inhibits lncXIST-induced PDGRA expression and M2 macrophage polarization. (**A**) qRT-PCR analysis of lncXIST levels in HCT116 cells after siRNA knockdown. (**B**) Schematic diagram of si-lncXIST HCT116 cells co-cultured with THP-1 (Mφ) cells using the Transwell system. (**C**) qRT-PCR was performed to detect the expression levels of M2 markers CCL22, IL10, and PDL1 in THP-1(Mφ) cells treated with PBS, HCT116 cells, si-NC HCT116 cells, and si-lncXIST HCT116 cells. (**D**) Predicted binding sites between miR-17-5p (wild-type WT or mutant MUT), PDGFRA, and lncXIST. Nucleotides highlighted in red represent mutant binding sites. (**E**,**F**) Dual-luciferase reporter assay showed miR-17-5p interacted with (**E**) PDGFRA-WT and (**F**) XIST-WT at the predicted sites in 293T cells. (**G**) qRT-PCR analyses showed reduced lncXIST, and increased miR-17-5p and PDGFRA levels were found in si-lncXIST knockdown THP-1(Mφ) cells. (**H**) qRT-PCR analyses showed increased lncXIST, and reduced miR-17-5p and PDGFRA levels were found in ov-lncXIST THP-1(Mφ) cells. (**I**) Transfection of miR-17-5p mimics in THP-1 (Mφ) cells showed increased expression level of miR-17-5p and decreased expression level of PDGFRA. (**J**) Simultaneous overexpression of lncXIST and miR-17-5p in THP-1(Mφ) cells was performed. qRT-PCR results showed that the expression level of lncXIST was significantly decreased, the expression level of miR-17-5p was significantly increased, and the expression level of PDGFRA was significantly reduced in the over-XIST+miR-17-5p mimic group, compared with the control group. (**K**) Expression of M2 phenotypic markers CCL22, IL10, and PDL1 in THP-1 (Mφ) cells were detected by qRT-PCR. Compared with the control group, the over-XIST group significantly increased the expression of CCL22, IL10, and PDL1. Compared with the over-XIST+NC mimic group, the over-XIST+miR-17-5p mimic group showed significantly reduced CCL22, IL10, and PDL1 expression levels. All experiments were performed in triplicates, ns denotes no-significance, * denotes *p* < 0.05, ** denotes *p* < 0.01, *** denotes *p* < 0.001, and **** indicates *p* < 0.0001.

To examine the function of lncXIST in shaping macrophage phenotype, we first knocked down lncXIST in HCT116 cells and co-cultured HCT116-siXIST cells with THP-1(Mφ) cells (Figure 5A,B). The qRT-PCR results demonstrated that while THP-1(Mφ) cells co-treated with HCT116-NC cells showed increased M2 macrophage markers (CCL22, IL10, and PDL1), treatment with HCT116-siXIST cells inhibited M2 markers (Figure 5C). These results suggest that knockdown of lncXIST reduces the effect of HCT116 cells on M2 macrophage polarization. To test whether lncXIST is able to regulate PDGFRA expression by competitive adsorption of miR-17-5p, we first confirmed the binding interaction via dual-luciferase reporter gene assay. The results showed that overexpression of miR-17-5p significantly reduced the luciferase activity of wild-type PDGFRA and wild-type lncXIST binding site vectors but not mutant binding site vectors in 293T cells (Figure 5D–F). Moreover, to determine the effect of lncXIST on the expression of miR-17-5p and PDGFRA, we knocked down and overexpressed lncXIST in THP-1 (Mφ) cells. In lncXIST knockdown THP-1 (Mφ) cells, miR-17-5p was significantly upregulated, while PDGFRA mRNA levels were downregulated (Figure 5G). In contrast, overexpressing lncXIST led to reduced miR-17-5p and elevated PDGFRA mRNA levels (Figure 5H). Moreover, miR-17-5p mimics were transfected in THP-1(Mφ) cells to determine the effect of miR-17-5p on PDGFRA expression. As indicated in Figure 5I, PDGFRA mRNA levels were significantly upregulated in the miR-17-5p mimic group compared to the NC mimic group. These results suggest that lncXIST can adsorb miR-17-5p and, in turn, promote PDGFRA mRNA expression. In addition, to determine the rescue effect of miR-17-5a, lncXIST overexpression plasmid and miR-17-5p mimic were co-transfected in THP-1 (Mφ) cells. As shown in Figure 5J, overexpressing lncXIST inhibited miR-17-5p and promoted PDGFRA mRNA levels. Interestingly, compared to the over-XIST+NC mimic group, overexpressing both lncXIST and miR-17-5p led to increased miR-17-5p and reduced PDGFRA levels (Figure 5J). These results suggest that miR-17-5p is capable of reversing the effect of lncXIST on PDGFRA expression levels. Lastly, qRT-PCR results showed that compared with the control group, the over-XIST group demonstrated significantly increased expression of M2 phenotypic markers CCL22, IL10, and PDL1 (Figure 5K). On the other hand, the over-XIST+miR-17-5p mimic group showed significantly reduced M2 markers compared with the over-XIST+NC mimic group (Figure 5K). This suggests that miR-17-5p not only hinders the impact of lncXIST on PDGFRA levels, but also its effect in promoting M2 macrophage polarization.

## 3. Discussion

The tumor microenvironment is a tumor-permissive milieu orchestrated by malignant tumor cells to facilitate continued growth and metastasis. Macrophages are integral components of the innate immune system and constitute a significant fraction of the TME. In fact, macrophages account for up to 50% of the mass in solid tumors [27]. Macrophages exhibit high plasticity and heterogeneity and can be modulated by tumor cells to polarize into tumor-promoting M2 phenotype. As efficient cargos for promoting cell–cell communication, it has become increasingly evident that tumor-derived exosomes are abundant in the TME and play an important role in maintaining an immunosuppressive microenvironment. A growing body of research suggests that tumor cell-derived exosomes can promote tumor development by inducing macrophage M2 polarization [28,29,30,31,32]. While most studies have focused on the effects of exosome-encapsulated miRNAs on macrophages, research on the effect of tumor derived exosomal lncRNAs remains scarce, particularly in the context of colon cancer. In this study, we focused on identifying the role of colon cancer exosomal lncRNAs on promoting macrophage polarization.

First, we confirmed that colon cancer cells retain the capacity to induce M2 macrophage polarization. During our investigation, we observed that THP-1 (Mφ) cells co-cultured with either CT26 or HCT116 cells demonstrated increased M2 macrophage phenotypic markers including CCL22, IL10, and PDL1. However, treatment with GW4869, a commonly used exosome inhibitor, reversed these effects. This finding led us to postulate that exosomes may act as crucial mediators in remodeling the macrophage phenotype. To verify this hypothesis, we isolated exosomes from CT26 and HCT116 cells through ultracentrifugation, and co-cultured them with THP-1 (Mφ) cells. Our results indicated that THP-1 (Mφ) cells treated with tumor-derived exosomes showed significantly increased M2-related proteins including ARG1, TGF-β, PDL1, and CD206. In contrast to M2 markers, M1 markers such as INOS and CD86 were either unchanged in the CT26-exo group, or significantly reduced in the HCT116-exo group. These results unequivocally confirm that colon cancer cell-derived exosomes have the ability to induce macrophages to polarize toward the M2 phenotype. Similar phenomena have been reported in other tumors, including bladder, breast, lung, and pancreatic cancers [22,33,34,35]. To examine the effect of polarized macrophages on colon cancer progression, medium from CT26-exo- and HCT116-exo-treated THP-1 (Mφ) cells was collected and used to treat CT26 and HCT116 cells. As a result, the rates of proliferation, migration, and invasion were significantly enhanced, indicating that M2 macrophages induced by tumor cell-derived exosomes can, in turn, enhance tumor cell growth. Interestingly, Lin et al. has reported that exosomes derived from bladder cancer cells polarized macrophages to the M2 phenotype, which, in turn, promoted migration and invasion of bladder cancer cells [22]. Moreover, Chen et al. found that exosomes from adriamycin-resistant breast cancer cells induced M2 macrophage polarization to mediate proliferation, migration and invasion of breast cancer cells [14]. These studies further demonstrate the reliability of the present findings.

Having confirmed the ability of colon cancer-derived exosomes to promote M2 macrophage polarization, we set out to explore the underlying mechanism. RNA-seq followed by KEGG and GSEA enrichment analyses were performed on HCT116-exo-treated THP-1 (Mφ) cells. Through Western blotting analyses, we confirmed that HCT116-exo and CT26-exo simultaneously promoted the activation of three M2 macrophage-related signaling pathways including PI3K/AKT, MAPK/ERK/ERK, and JAK-STAT in THP-1 (Mφ) cells. Although all three pathways have been separately demonstrated to be implicated in M2 macrophage polarization, the activation of all three at the same time has not been previously reported. A study led by Lin et al. showed that bladder cancer cell-derived exosomes can activate the PI3K-AKT pathway to induce M2 polarization [22]. Qiu et al. found that gastric cancer cell-derived exosomes are able to induce M2 macrophage polarization by activating the MAPK/ERK/ERK signaling pathway [26]. In addition, Chen et al. showed that exosomes from lung adenocarcinoma cells could polarize macrophages toward the M2 phenotype by activating STAT3 [14]. Moreover, Cai et al. demonstrated that STAT6 was an important factor in M2 polarization by oral squamous cell carcinoma-derived exosomes [25]. In the present study, we hypothesize that colon cancer cell-derived exosomes may activate multiple pathways in the process of promoting M2 macrophage polarization. By screening for common DEGs between these three signaling pathways, we found that PDGFRA was most significantly upregulated in CT26-exo/HCT116-exo-treated THP-1 (Mφ) cells, both at the mRNA and protein levels. PDGFRA encodes the platelet-derived growth factor (PDGF) receptor α protein. When activated by PDGF, PDGFRA can, in turn, initiate key downstream PI3K-AKT, MAPK/ERK/ERK, and JAK-STAT pathways to regulate cell proliferation, growth, and differentiation [36,37]. Aberrant activation of PDGFRA has been reported in gastrointestinal mesenchymal tumors, glioma tumors, and gastric cancer [38]. However, the role of PDGFRA in M2 macrophage polarization remain underexplored.

To explore potential exosome-encapsulated upstream regulators of PDGFRA, we first screened for potential target miRNAs using miRtarbase, TargetScan, miRDB, Tarbase, and Pic Tar databases. Subsequent qRT-PCR validation revealed that miR-17-5p expressions were significantly reduced in CT26-exo- and HCT116-exo treated THP-1 (Mφ) cells. Furthermore, through Starbase, miRcord, as well as colon cancer GEO databases (GSE186577, GSE84983, and GSE104364), we pinpointed to lncFGD5-AS1, lncSNHG14, and lncXIST as potential miR-17-5p-targeting lncRNAs. Verification via qRT-PCR indicated that lncXIST was highly expressed in colon cancer cells. Moreover, lncXIST was found encapsulated and highly enriched in colon cancer cell-derived exosomes, as well as colon cancer-derived exosome-treated THP-1 (Mφ) cells. In addition, lncXIST knockdown HCT116 cells were co-cultured with THP-1 (Mφ) cells. The results indicated that knockdown of lncXIST significantly reversed M2 macrophage polarization. Meanwhile, dual-luciferase reporter gene assay also confirmed the direct interaction between lncXIST and miR-17-5p, as well as between miR-17-5p and PDGFRA. Based on the above results, this study conjectured that lncXIST was transferred from colon cancer cells to THP-1 (Mφ) cells via exosomes. In addition, lncXIST could sponge miR-17-5p and, thereby, enhance PDGFRA expression and promote M2 macrophage polarization. To verify this, we knocked down and overexpressed lncXIST in THP-1(Mφ) cells and observed changes in miR-17-5p and PDGFRA levels by qRT-PCR. Results showed that the knockdown of lncXIST led to an increased expression of miR-17-5p and a reduced expression of PDGFRA. On the contrary, the overexpression of lncXIST inhibited miR-17-5p, but promoted PDGFRA expression. Furthermore, we overexpressed both lncXIST and miR-17-5p in THP-1 (Mφ) cells and found that elevated miR-17-5p expression inhibited PDGFRA expression as well as the degree of M2 macrophage polarization. Altogether, our data suggest that lncXIST from colon cancer-derived exosomes promotes PDGFRA expression by inhibiting miR-17-5p.

miR-17-5p is a member of the miR-17-92 cluster, a family whose organization and sequence are highly conserved among vertebrates, and whose evolutionary process has produced two mammalian paralogs due to gene duplication and deletion events: the miR-106b-25 cluster and the miR-106a-363 cluster. This means that miR-106 is a homolog of miR-17 [39]. miR-17-5p [40] is an important regulator of cell proliferation, autophagy and apoptosis, and plays an important regulatory role in cancer [41]. Xu et al. found that lncNEAT1 promotes angiogenesis in gastric cancer by sponging miR-17-5p to increase the expression of TGF-βR2. Chen et al. found that lncPART1 promotes non-small cell carcinoma by sponging miR-17-5p to increase the expression of TGF-βR2 expression to promote the progression of non-small cell lung cancer cells [42]. In addition, Xu et al. found that lncMIR17HG promotes colon cancer progression by sponging miR-375 to increase the expression of NF-κB/RELA [43]. lncXIST is known for mediating transcriptional silencing of genes on the X chromosome and acts as a dosage compensation between male and female chromosomes, but it still has a developmental role in various diseases, including stroke, inflammation, cardiomyocyte hypertrophy, and cancer [44,45]. lncXIST was found to be involved in the development of various types of cancer and is dysregulated in tumor cells, promoting tumor proliferation, migration, angiogenesis, and chemotherapy resistance [46,47,48]. Wei et al. found that lncXIST is upregulated in pancreatic cancer tissues and cell lines and participated in promoting EGFR expression by sponging miR-133a to promote pancreatic cancer proliferation [49]. Liu et al. found that lncXIST was upregulated in thyroid cancer tissues and cell lines and promoted the activation of the MET-PI3K-AKT signaling pathway through sponging miR-34a to promote the development of thyroid cancer [50]. LncXIST was also found to be upregulated in colon cancer tissues and cell lines. Specifically, Sun et al. found that lncXIST activated the Wnt/β-catenin pathway by sponging miR-34a to promote colon cancer development [21]. Yang et al. found that METTL14 inhibited colorectal cancer development by suppressing lncXIST [51]. In addition, lncXIST is also closely related to exosomes. Cheng et al. found that lncXIST-containing exosomes secreted by pancreatic cancer cells increased the expression of GDNF to promote perineural infiltration of pancreatic cancer cells by sponging miR-211-5p [52]. Overall, these studies provide additional support for the reliability of the present finding, that lncXIST can be carried by exosomes and act as sponges for target miRNAs to promote colon cancer development.

Altogether, the results from this study demonstrated that exosomal lncXIST can induce macrophage M2 polarization by regulating the miR-17-5p/PDGFRA axis to promote colon cancer progression. However, there are some limitations of this study. First, this study mainly used colon cancer cell lines for in vitro experiments, while subsequent validation in more colon cancer cell lines and in vivo animal experiments are needed. Second, our data only demonstrated that the upregulation of PDGFRA promoted M2-like macrophage polarization, but not its activation. Further validation is required. Third, while PDGFRA was a common gene found for the AKT-, ERK-, and STAT3/6 signaling pathways, the effect of the lncXIST/miR-17-5p/PDGFRA axis on the activation of each pathway requires further examination. Fourth, the specific mechanisms by which M2-like macrophages promote colon cancer cell proliferation, migration, and invasion have not been explored in this study. To elaborate on this section, the ability of CT26 and HCT116 cells to proliferate, migrate, and invade needs to evaluated when co-cultured with culture medium from M2-like macrophages. Moreover, the levels of lncXIST, miR-17-ap, and PDGFRA should also be evaluated in the treated cancer cells. Furthermore, it is noteworthy that the repertoire of bioactives encapsulated in colon cancer-derived exosomes extends beyond lncXIST, potentially harboring additional factors that contribute to the induction of macrophage M2 polarization and, subsequently, facilitate colon cancer progression. These unexplored bioactives represent intriguing avenues for future research, as they may hold the key to unraveling novel mechanisms underlying the intricate interplay between the tumor microenvironment and cancer development. Therefore, further investigation into these exosomal contents is warranted to gain a more comprehensive understanding of their roles in modulating macrophage polarization and promoting colon cancer progression, ultimately informing the development of targeted therapeutic strategies.

## 4. Materials and Methods

### 4.1. Cell Lines and Cell Culture

CT26, HCT116, NCM460, and 293T cells were purchased from the Chinese Academy of Sciences CellBank (Shanghai, China). THP-1 (human monocytic-leukemia cells) cells were purchased from Wuhan Prolife Technology Co. (Wuhan, China). CT26 and HCT116 cells were cultured in RPMI-1640 medium (Hyclone) with 10% fetal bovine serum (GIBCO BRL, Grand Island, NY, USA) and 1% penicillin. NCM460 and 293T cells were cultured in DMEM high glucose medium (GIBCO BRL, Grand Island, NY, USA) with 10% Newzerum fetal bovine serum (NEWZERUM Ltd., Christchurch, New Zealand)and 1% penicillin. THP-1 cells were cultured using THP-1 complete medium (Wuhan Pricella Biotechnology Co., Ltd., Wu Han, China). All cells were maintained at 37 °C containing 5% CO_2_.

### 4.2. Macrophage Induction from Monocytes

At approximately 80–90% confluency, THP-1 cells were centrifuged and the supernatant was aspirated, and the cells were resuspended using 1 mL β-mercaptoethanol-free THP-1 complete medium. After counting, 10^6^ cells were taken and inoculated in a single well of a 6-well plate, followed by the addition of 2 μL of 100 μg/mL phorbol-12-myristate-13-acetate (PMA; Sigma-Aldrich, St. Louis, MO, USA) (final concentration of 100 ng/mL) for induction. THP-1 cells were fully differentiated into macrophages (Mφ) after 48 h.

### 4.3. Co-Culturing System

To mimic exosome-mediated intercellular communication between colon cancer cells and TAMs, an in vitro indirect co-culture system was established in which macrophages and colon cancer cells were inoculated in the upper and lower chambers of transwell cell culture inserts with polycarbonate membranes, respectively. After 48 h, the cells were harvested.

### 4.4. Cell Transfection

Transfection was carried out using jetPRIME buffer (Polyplus-transfection SA, Illkirch, France) according to manufacturer’s protocol. Plasmids and siRNAs were designed and constructed by GenePharma (Shanghai, China). Sequence information is listed in Supplementary Table S2.

### 4.5. Extraction of Exosomes

CT26 and HCT116 cells were cultured using complete medium prepared with exosome-free serum; cell culture medium was collected into 50 mL centrifuge tubes when the cell coverage reached 90%. The cells were then centrifuged at 4 °C for 5 min at 800× *g*, and again for 10 min at 2000× *g*. The supernatant was aspirated with a syringe and paired with a 0.22 μm filter for filtration, and the filtered supernatant of the cell culture medium was transferred to a sterile Beckman centrifuge tube and centrifuged in a Beckman ultracentrifuge (BeckmanCoulter, Miami, FL, USA) with the parameters of 36,900 speed (100,000× *g*) at 4 °C for 2 h. After centrifugation, the tubes were transferred to an ultraclean bench, where the supernatant was discarded, and exosomes were resuspended with PBS.

### 4.6. Transmission Electron Microscopy (TEM)

First, 10 μL of exosome suspension was added dropwise onto a copper mesh, and let stand for 1 min. The floating liquid was then absorbed using filter paper. Next, 10 μL of 1% phosphotungstic acid staining solution was added for staining. After 5 min, excess liquid was absorbed using filter paper. Then, 10 μL of purified water was added dropwise and absorbed with filter paper in order to remove the excess dye. After drying at room temperature, the morphology of exosomes was then observed and photographed using transmission electron microscopy (Thermo FisherScientific, Waltham, MA, USA).

### 4.7. Flow Cytometry of Exosomes

First, 10 μL of exosome suspension was withdrawn and subjected to serial dilutions using a flow-through sheath solution. Various concentrations of the diluted exosome suspension were then introduced into the detection system to ascertain the optimal particle count. The optimal dilution concentration was meticulously determined based on the real-time monitoring of particle numbers (less than 10,000 particles). Subsequently, the exosome suspension at the determined concentration was evenly apportioned into 3 distinct centrifuge tubes. One tube was labeled blank, the second and third tubes were labeled PE-CD81 and PE-CD63, respectively. These samples were then incubated under standardized conditions of 15 min at room temperature, ensuring adequate protection from light exposure. Following incubation, the prepared samples were analyzed using a CytoFLEX flow cytometer (Beckman Coulter, Miami, FL, USA) for further characterization and analysis.

### 4.8. Exosome Labeling

Exosomes were labeled using the PKH26 Infrared Fluorescent Cell Crosslinker Kit (Sigma-Aldrich, St. Louis, MO, USA). First, 50 μL of the exosome suspension and 250 μL of Diluent C solution were gently mixed by pipetting in a centrifuge tube. In a separate centrifuge tube, 5 μL of PKH26 dye and 250 μL of Diluent C solution were gently mixed by pipetting. The mixtures from the two centrifuge tubes were then combined and gently mixed by pipetting. The resulting mixture was incubated at room temperature for 5 min to allow for efficient labeling. To terminate the reaction, 2 mL of 5% BSA was added at the end of the incubation period. Finally, the labeled exosomes were subjected to ultracentrifugation and resuspended in 50 μL of PBS for further analysis.

### 4.9. Fluorescence Confocal Microscopy

THP-1 cells were seeded onto cell crawls positioned in 24-well plates and, subsequently, differentiated into macrophages by treating with 0.5 μL of 100 μg/mL PMA for 48 h. Following differentiation, the cells were further incubated for 24 h in the presence of PKH26-labeled exosomes (red). Subsequently, 5 μL of Hoechst33342 live cell staining solution (blue) was added to each well containing 500 μL of medium, followed by incubation at 37 °C for 10 min. At the end of the incubation, the dye-containing medium was discarded and cells were washed twice with PBS. Cytoskeleton staining was performed using β-tubilin (green). The cells were fixed with 500 μL of 4% paraformaldehyde for 15 min, followed by 3 washes with PBS. Each well was then incubated with 500 μL of PBS containing 0.1% Triton X-100 for 2 min to permeabilize the cells. The cells were then blocked at room temperature for 40 min using skim milk powder on a shaker. The cells were then washed and incubated with primary β-tubilin antibody diluted in blocking solution overnight at 4 °C. Following incubation, the cells were then washed and incubated with Alexa Fluor^®^ 488 Donkey Anti-Mouse IgG (H + L) secondary antibody diluted in skimmed milk powder for 1 h at room temperature in the dark. The cell crawls were then removed and mounted on clean slides using fluorescent sealer (NaHCO3 3.7 g, anhydrous Na_2_CO_3_ 0.6 g dissolved in 100 mL ddH_2_O and mixed 1:1 with glycerol). Finally, the slides were allowed to dry completely before being analyzed using the fluorescence confocal microscopy (Leica, Wetzlar, Germany).

### 4.10. Western Blotting Analysis

Total cellular and exosomal protein samples were obtained using Radioimmunoprecipitation Assay (RIPA) Lysis and Extraction Buffer supplemented with a mixture of protease and phosphatase inhibitors. The resulting lysates were quantified using Bradford’s reagent (Shanghai Beyotime Biotechnology Co., Ltd., Shanghai, China). Protein samples were separated by 10% SDS-PAGE and transferred onto a polyvinylidene difluoride (PVDF) membrane. The membrane was then blocked in 5% skim milk for 1h at room temperature followed by incubation with primary antibody at 4 °C overnight. Following incubation, the membrane was washed three times with TBST and subjected to secondary antibody incubation for 1 h at room temperature. Protein bands were detected and analyzed under chemiluminescence (Bio-Rad, Hercules, CA, USA).

### 4.11. Reagents and Antibodies

DMSO and GW4869 were purchased from Sigma (Sigma, USA) and MedChemExpress (MedChem Express, Monmouth Junction, NJ, USA), respectively. PE-CD63 and PE-CD81 antibodies were purchased from Abgent (San Diego, CA, USA). p-AKT(Thr308), p-AKT(Ser473), and p-ERK antibodies were purchased from Cell Signaling Technology (Danvers, MA, USA). p-STAT3, STAT3, p-STAT6, STAT6, CD63, CD81, and TSG101 primary antibodies were purchased from Santa Cruz Biotechnology (Santa Cruz, CA, USA). Alexa Fluor^®^488 donkey anti-mouse IgG (H + L) secondary antibody was purchased from Jackson Immunoresearch Laboratories (West Grove, PA, USA). Calnexin, PDGFRA, and GAPDH antibodies were purchased from Shanghai PoWan Biotechnology Co. (Shanghai, China). β-Tubulin, AKT, and ERK antibodies were purchased from Wuhan Sanying Biotechnology Co. (Wuhan, China). ARG1, TGF-β, PDL1, and CD206 antibodies were purchased from Hangzhou Jingjie Bioscience and Technology Co., Ltd. (Hangzhou, China).

### 4.12. Quantitative Real-Time Polymerase Chain Reaction (qRT-PCR)

Total RNA was extracted using TRIGene reagent (Genestar, Beijing, China). miRNA was extracted using Exosomal RNA Isolation Kit (Norgen Biotek, Thorold, Ontario, Canada). DNase I, amplification grade (Invitrogen) was used to digest DNA contaminants. The concentration and quality of RNA samples were detected using microspectrophotometer Nanodrop 1000 (Thermo Fisher Scientific, Waltham, MA, USA). Complementary DNA (cDNA) synthesis of mRNA was performed using a reverse transcription kit Hifair^®^ II 1st Strand cDNA Synthesis SuperMix for Qpcr (Yeasen, Shanghai, China) and miRNA was performed using a reverse transcription kit StarScript III RT Kit (Genestar, Beijing, China). SYBR Green PCR premix (Epizyme, Shanghai, China) was used for qRT-PCR on a CFX96 Touch real-time fluorescent quantitative PCR detection system (Bio-Rad, USA). Relative level of gene expression was calculated using the 2^−ΔΔCt^ method. qRT-PCR primer (Sangon Biotech, Shanghai, China) sequences are shown in the Supplementary Table S1.

### 4.13. MTS Proliferation Assay

Cell proliferation was analyzed through MTS analysis. First, 2.5 × 10^4^ cells were cultured in 96-well plates under treatment for 24 h. Then, 10 μL of MTS was added to each well and incubated in the dark at room temperature for 2 h. The absorbance was measured at 490 nm using a colorimeter(Omega Biotech, Doraville, GA, USA).

### 4.14. Transwell Assay

Cells were digested, centrifuged, and resuspended with serum-free medium. CT26 and HCT116 cells were plated in transwell chambers containing serum-free medium. In the lower chamber of a 24-well plate, 400 μL of complete medium culture medium was added to each well, to which an additional 200 μL of CT26-exo/HCT116-exo-treated macrophage medium was added to the experimental group and 200 μL of PBS-treated macrophage medium was added to the control group. Using 500 μL of 4% paraformaldehyde, the cells were placed into the chambers and fixed for 15 min. Paraformaldehyde was aspirated and discarded, and 0.1% crystal violet staining solution was added for 30 min, followed by imaging under a microscope. The invasion assay needs to be pre-lined with organoGel (Guangzhou Ebix Biotechnology Co., Ltd., Guangzhou, China) in the invasion experiments.

### 4.15. Dual-Luciferase Reporter Assay

Fluorescein-labeled reporter gene detection was assessed using Dual-luciferase Assay (Shanghai Beyotime Biotechnology Co., Ltd., Shanghai, China). The sequences were constructed in pmirGLO plasmid by Beijing Prime Biotechnology Co. (Beijing, China). First, 293T cells were seeded in 96-well plates and cultured for 24 h. The cells were then co-transfected with either wild-type or mutant vectors with NC or miR-17-5p mimics. After a 48h incubation period, passive lysis buffer was added to each well, and the supernatant was extracted for further analysis. Lysis buffer sample (40 μL) and firefly fluorescein lyase assay solution (100 μL) were mixed and added to each well of the 96-well plate. Absorbance was measured at 580 nm using a microplate reader (Omega, Germany). Sea kidney fluorescinase assay working solution (100 μL, sea kidney fluoresceinase assay substrate: sea kidney fluoresceinase assay buffer = 1:100) was then added to each well and the absorbance was measured at 460 nm.

### 4.16. Bioinformatics Analysis

miRtarbase (https://mirtarbase.cuhk.edu.cn/~miRTarBase/miRTarBase_2022/php/index.php), TargetScan (https://www.targetscan.org/vert_80/), miRDB (https://mirdb.org/), Tarbase (https://dianalab.e-ce.uth.gr/html/diana/web/index.php?r=tarbasev8), and Pic Tar (https://pictar.mdc-berlin.de/) databases were used to screen miRNA-targeting genes. The Starbase (https://starbase.sysu.edu.cn) and miRcord (http://www.mircode.org) databases were used to jointly predict lncRNAs targeting miR-17-5p. Colon cancer-related lncRNAs were screened using the GEO (GSE186577, GSE84983, and GSE104364) (https://www.ncbi.nlm.nih.gov/geo) database. All databases were accessed between 1–7 August 2023.

### 4.17. Statistical Analysis

The data were statistically analyzed and graphed using GraphPad Prism 8.0. The luminescence results of Western blotting bands were analyzed using ImageJ (Version 1.53m). *T*-test was used to analyze two independent samples with normal distribution, and one-way ANOVA test was used to comparing multiple samples. Multiple *t*-tests (and non-parametric tests) were used for comparing 2 independent samples with multiple data sets. Experiments were repeated independently 3 times. NS indicates no significance, * indicates *p* < 0.05, ** indicates *p* < 0.01, *** indicates *p* < 0.001, **** indicates *p* < 0.0001.

## 5. Conclusions

In conclusion, our study sheds light on the crosstalk between colon cancer-derived exosomes and macrophages. Our results underscore the novel role of lncXIST in promoting M2-like macrophage polarization via the miR-17-5a/PDGFRA axis as well as its potential as both a diagnostic biomarker and a therapeutic target for colon cancer. However, important questions remain to be explored. For one, as an X-linked gene, does the activation or inactivation of lncXIST have varied roles in male and female colon cancer patients? Moreover, in addition to macrophage polarization, is lncXIST involved in other pathways that may synergistically contribute to colon cancer development? Further investigation into the role of lncXIST in facilitating early diagnosis and advancing the treatment landscape is warranted.

## Data Availability

Data are available upon request.

## Supplementary Materials

The following supporting information can be downloaded at: https://www.mdpi.com/article/10.3390/ijms252111433/s1.
